# Functional gradient dysfunction in drug-naïve first-episode schizophrenia and its correlation with specific transcriptional patterns and treatment predictions

**DOI:** 10.1017/S0033291724001739

**Published:** 2024-11

**Authors:** Guanqun Yao, Jing Luo, Jing Li, Kun Feng, Pozi Liu, Yong Xu

**Affiliations:** 1 Department of Psychiatry, First Hospital/First Clinical Medical College of Shanxi Medical University, Taiyuan, 030001, China; 2 School of Medicine, Tsinghua University, Beijing, 100084, China; 3 Department of Rheumatology, The Second Affiliated Hospital of Zhejiang University School of Medicine, Hangzhou, 310000, China; 4 College of Humanities and Social Science, Shanxi Medical University, Taiyuan, 030001, China; 5 Shanxi Key Laboratory of Artificial Intelligence Assisted Diagnosis and Treatment for Mental Disorder, First Hospital of Shanxi Medical University, Taiyuan, China; 6 Department of Psychiatry, Yuquan Hospital, Tsinghua University, Beijing, 100040, China; 7 Department of Clinical Psychology, The Eighth Affiliated Hospital, Sun Yat-Sen University, Shenzhen, 518031, China

**Keywords:** cell type-specific signatures, cortical layers enrichment, first-episode schizophrenia, functional connectome gradient, transcriptional patterns, treatment outcomes

## Abstract

**Background:**

First-episode schizophrenia (FES) is a progressive psychiatric disorder influenced by genetics, environmental factors, and brain function. The functional gradient deficits of drug-naïve FES and its relationship to gene expression profiles and treatment outcomes are unknown.

**Methods:**

In this study, we engaged a cohort of 116 FES and 100 healthy controls (HC), aged 7 to 30 years, including 15 FES over an 8-week antipsychotic medication regimen. Our examination focused on primary-to-transmodal alterations in voxel-based connection gradients in FES. Then, we employed network topology, Neurosynth, postmortem gene expression, and support vector regression to evaluate integration and segregation functions, meta-analytic cognitive terms, transcriptional patterns, and treatment predictions.

**Results:**

FES displayed diminished global connectome gradients (Cohen's *d* = 0.32–0.57) correlated with compensatory integration and segregation functions (Cohen's *d* = 0.31–0.36). Predominant alterations were observed in the default (67.6%) and sensorimotor (21.9%) network, related to high-order cognitive functions. Furthermore, we identified notable overlaps between partial least squares (PLS1) weighted genes and dysregulated genes in other psychiatric conditions. Genes linked with gradient alterations were enriched in synaptic signaling, neurodevelopment process, specific astrocytes, cortical layers (layer II and IV), and developmental phases from late/mid fetal to young adulthood. Additionally, the onset age influenced the severity of FES, with discernible differences in connection gradients between minor- and adult-FES. Moreover, the connectivity gradients of FES at baseline significantly predicted treatment outcomes.

**Conclusions:**

These results offer significant theoretical foundations for elucidating the intricate interplay between macroscopic functional connection gradient changes and microscopic transcriptional patterns during the onset and progression of FES.

## Introduction

Schizophrenia (SCZ) is a progressive psychiatric disorder defined by significant disturbances in reality perception, emotional responses, and cognitive processes (Insel, 2010), involving various elements such as brain function, genetics, and environmental influences (Rantala, Luoto, Borráz-León, & Krams, 2022). First-episode SCZ (FES) typically manifests during the adolescence periods, and recurrent or prolonged psychotic episodes significantly impair neurological integrity, cognitive functions, and the overall prognosis of the patient (Ren et al., 2013; Zhang et al., 2020). While previous studies have underscored notable aberrations in brain functionality and therapeutic responses in FES, the underlying pathophysiological mechanisms remain elusive (Li et al., 2023). Investigating the neurobiological underpinnings of FES is crucial for early detection and intervention.

The hierarchical structure serves as the foundational organizing principle in the human brain, enabling effective information coding and integration from sensory perception to cognitive processing (Mesulam, 1998; Wang, Zhou, Ding, & Xiao, 2021). Functional connectivity and gradients collectively indicate the development of brain network hierarchies during neurodevelopment (Xue et al., 2023). These hierarchies are believed to channel sensory input through multiple cortical relays into cross-modal regions (Liu, Betzel, & Misic, 2022). Such an organizational structure is posited to facilitate the integration of cognitive processes, abstract concepts, and behaviors (Xia et al., 2022). In healthy subjects, the principal gradient extends from the primary sensory network to the transmodal default mode network (DMN), mirroring the hierarchical synaptic distribution observed in postmortem brains (Haueis, 2021). Given the concurrent abnormalities in sensory and cognitive processing in SCZ (Dondé et al., 2020; Hamilton et al., 2018), delineating the gradients from primary to transmodal in FES could offer insights into this prevalent deficit through the lens of brain hierarchy. Our previous study on thalamic functional connectivity gradients have reported heightened disjunction in macroscopic thalamic functional organization among early-onset SCZ (EOS), linked with modified thalamocortical interactions in both unimodal and transmodal networks (Fan et al., 2023). Additionally, analyses centered on the subcortical functional connectivity gradient have suggested that shifts in the limbic system gradient in FES might serve as a pertinent marker for drug therapy responsiveness (Yang et al., 2023). Although disruptions in functional gradient patterns within subcortical regions have been explored in FES, numerous studies have identified FES as a disorder indicative of global brain dysfunction (van Haren et al., 2020; Yang et al., 2014). However, the alterations in the whole-brain functional connectivity gradient of FES and their correlations with the brain's integration and segregation capacities, cognitive deficits, and treatment responses are yet to be fully elucidated.

Previous studies have highlighted the significant role of genes in human brain networks, primarily in shaping functionally crucial connections between network hubs (Arnatkeviciute et al., 2021). The whole genome-wide association studies (GWAS) have identified SCZ as a polygenic, complex psychiatric disorder with several risk loci and massive independent single-nucleotide polymorphisms contributing to high heritability (Ripke et al., 2013). The Allen Human Brain Atlas (AHBA) microarray dataset facilitates the identification of transcriptomes correlated with human neuroimaging, presenting multimodal evidence that suggest a relationship between conserved gene expression and functionally relevant neural circuits (Ritchie, Pantazatos, & French, 2018). Recently, the transcriptome-architecture association studies also offer new avenues for investigating potential relationships between macroscale architectural abnormalities and specific transcriptional expression patterns in FES (Morgan et al., 2019). However, the abnormalities of macroscale architecture in brain can't directly reflect the differences of functional connectomes in FES. Consequently, substantial studies have initiated investigations into the potential correlations between functional gradient dysfunctions and transcriptional profiles across various mental health conditions, including bipolar disorder (BD) (Lei et al., 2023) and major depressive disorder (MDD) (Xia et al., 2022). However, the combined evaluation of functional gradient differences and regional gene-expression patterns for FES has not yet been established. Further studies are required to explore potential pathogenesis and develop potential therapeutic targets for individuals with FES.

In this study, we investigated functional gradient anomalies in drug-naïve FES and examined their correlation with transcriptional expression profiles and treatment outcomes. Initially, we utilized the functional connectivity gradient to identify differences in primary-to-transmodal gradients in FES compared to healthy controls (HC). We then analyzed the association between gradient alterations, network topology, and meta-analytic cognitive terms. Using the AHBA dataset, we explored the connection between gradient shifts in FES and corresponding gene expression patterns. Additionally, Permutation tests were performed to assess the significance of shared genes between gradient changes in FES and published dysregulated genes in other psychiatric disorders. We further identified specific cell types to deduce their contribution to the transcriptomic relationship associated with gradient alterations in FES. Furthermore, we performed enrichment analyses focusing on special cortical layers and developmental stages to elucidate potential connections between transcriptomic anomalies, gradient alterations, and developmental characteristics in FES. Finally, we delved into the correlations between the age of onset and disease severity, and applied the support vector regression (SVR) model to assess the predictive capacity of the connectome gradient concerning treatment outcomes in FES.

## Methods

### Participants and imaging preprocessing

This study recruited 216 participants between the ages of 7 and 30, of which 116 were diagnosed with FES and evaluated by two psychiatrists affiliated with the First Hospital of Shanxi Medical University. The remaining 100 HC were sourced from neighboring communities. The diagnosis of FES was established by two professional psychiatrists following a structured clinical interview (SCID) for the Fourth Edition of the Diagnostic and Statistical Manual of Mental Disorders Structured Clinical Interview (DSM-IV) after at least a 6-month follow-up. Following the initial diagnosis, resting-state functional magnetic resonance imaging (rs-fMRI) scans were performed on all participants. Of the 116 FES, 12 lacked data on the Positive and Negative Symptom Scale (PANSS), a recognized metric for assessing SCZ severity (Koblan et al., 2020). Additionally, 15 FES patients consented to an 8-week follow-up, during which risperidone was prescribed at dosages of 4–6 mg/day, tailored according to the patient's weight, symptom severity, and response to treatment. Subsequently, the PANSS was re-evaluated for these 15 individuals at the end of the follow-up period.

The eligibility criteria for FES were defined by the following parameters: (i) aged between 7 and 30 years, right-handedness, and Han nationality; (ii) compliance with DSM-IV diagnostic criteria for FES; (iii) an initial FES diagnosis without previous exposure to psychotropic medications. Exclusion criteria encompassed: (i) coexistence of Axis-I or Axis-II disorders; (ii) FES duration surpassing one year; (iii) manifest severe organic brain or systemic diseases; (iv) any history of claustrophobia or possession of metal implants. Furthermore, HC exhibited no mental disorders or family history of mental illness. Ethical approval for this research was obtained from the Ethics Committee of the First Hospital of the Medical University. All participants and their guardians provided informed consent through their signatures. Demographic and clinical characteristics were detailed in online Supplementary Table S1. Details on imaging acquisition and preprocessing were available in the online Supplementary material.

### Voxel-based connectome gradient construction

To diminish computational complexity, we resampled the preprocessed functional images to a uniform 4-mm isotropic resolution, encompassing 18 933 voxels. We initially built a voxel-wise functional connectome matrix comprising 18 933 nodes for each participant. Subsequently, the diffusion map embedding techniques were used for determining the connectome gradient (Margulies et al., 2016). Specifically, we retained the top 10% of connection for each node and computed the cosine similarity across all pairs of nodes. This similarity matrix was transformed into a normalized angle matrix to circumvent negative values (Larivière et al., 2020). The diffusion map embedding was then employed to discern gradient component that elucidated the variations within the functional connectome pattern. Using iterative Procrustes rotation, the resulting gradient maps were harmonized across participants (Hong et al., 2019). Recognizing that the principal gradient is intrinsically linked to cortical microstructure and cognitive competence (Huntenburg, Bazin, & Margulies, 2018), our subsequent emphasis was on FES-related differences in the first gradients. Moreover, we calculated three global gradients: gradient range, explanation ratio, and variation.

Differences in the connectome gradient between FES and HC were evaluated through a general linear model (GLM), which accounted for age and sex as covariates. Then, two-sided *t* tests (contrast = FES-HC) were performed. Additionally, to delve deeper into the impact of sex on gradient disparities between groups, an auxiliary GLM was used to inspect potential sex-by-group interactions. For global gradients, the significance threshold was established using the false discovery rate (FDR) correction of *p* < 0.05. Meanwhile, for the regional gradient maps, the threshold was delineated at *p* < 0.001 at the voxel level and subsequently adjusted with a Gaussian random field (GRF) correction to *p* < 0.05 at the cluster level.

### Association of network topology with functional gradients in FES

We computed the area under the curve (AUC) for normalized characteristic shortest path length (aLambda), normalized clustering coefficient (aGamma), and small-world network (aSigma) for each participant's whole-brain networks. Importantly, aGamma measures brain network segregation, while aLambda evaluates network integration (Gao et al., 2023). Additionally, aSigma quantifies the equilibrium between integration and segregation functions (Bullmore & Sporns, 2009). A detailed description of the network topology construction was provided in the online Supplementary material. Subsequently, we assessed between-group differences in network topology using a GLM, with age and gender as covariates. Furthermore, we also investigated the correlations between global gradients and network topology. The significance threshold was established by the FDR correction of *p* < 0.05.

### Association of meta-analytic cognitive terms with gradient alterations in FES

The Neurosynth (https://neurosynth.org/) was utilized to investigate the correlations between meta-analytic cognitive terms and gradient changes in FES (Yarkoni, Poldrack, Nichols, Van Essen, & Wager, 2011). The threshold *z* maps, stemmed from regional gradient comparisons, were categorized into FES-positive and FES-negative maps. Then, the ‘decoder’ function in Neurosynth was employed to evaluate spatial correlation between maps associated with FES-negative and FES-positive conditions and the meta-analytic maps corresponding to each cognitive term within the database. The significance of these correlation coefficients was ascertained through Permutation tests, accounting for spatial autocorrelations (Burt, Helmer, Shinn, Anticevic, & Murray, 2020). The detailed description was provided in the online Supplementary material.

### Transcriptional profiles obtainment and preprocessing

The AHBA database was utilized to acquire gene expression profiles from six postmortem brain tissues (http://human.brain-map.org) (Hawrylycz et al., 2012). Due to restricted availability of samples of the right hemisphere (only two brains) in the AHBA datasets, transcriptional profiles of the left hemisphere were selected for our subsequent analysis. Then, we applied the Python toolbox (abagen) to process the AHBA dataset and map the transcriptional data onto 180 brain gradients within the homologous left hemisphere (https://github.com/rmarkello/abagen) (Markello et al., 2021). The standardized preprocessing procedure was performed based on a recommended protocol with seven steps, including conversing gene symbols, filtering low-intensity data, selecting homologous probes, addressing missing data, mapping samples to brain gradients, normalizing the samples, and identifying reliable genes (Arnatkeviciute, Fulcher, & Fornito, 2019). Consequently, this process yielded a gene expression matrix encompassing 180 brain gradients and 15 631 genes.

### Correlation analysis between transcriptional signatures and gradient abnormities in FES

The partial least squares (PLS) regression was used for investigating correlations between gene expression and gradient abnormities in FES, setting the expressional levels of all 15 631 genes as independent variables and the case–control *z* maps from 180 gradients as dependent variables (Abdi & Williams, 2013). The first or second PLS component (PLS1 or PLS2) is typically identified as the optimal low-dimensional interpretation of the covariance within the high-dimensional data matrix. Subsequently, we performed the spatial autocorrelation analysis with a Permutation test involving 10 000 iterations to examine whether the explained covariance between the gradient *z* statistic maps and PLS scores exceeded the expectation by chance. Additionally, the bootstrapping method, consisting of 10 000 bootstrap samples, was utilized to evaluate the variability of each gene within the PLS component. The Z values were calculated by dividing each gradient's expression weight by its bootstrap standard error, and all genes were then ranked to identify significant weighted genes with FDR-corrected *p* < 0.05, based on their weights to PLS component (Xue et al., 2023). To gain further insights into the functional signatures of these significant PLS+/− genes, we employed the Metascape software to perform various enrichment analyses (Zhou et al., 2019).

To explore potential correlations between transcriptional abnormalities in other psychiatric disorders and abnormal gradients in FES, we performed an analysis by overlapping the PLS+/− genes with the dysregulated genes of six brain-disorder diseases obtained from Gandal's study (Gandal et al., 2018). These diseases included adult-SCZ, MDD, inflammatory bowel disease (IBD), alcohol abuse disorder (AAD), BD, and autism spectrum disorder (ASD). Dysregulated genes were selected based om differential gene expressions **(**DGEs) >0 and FDR-corrected *p* < 0.05. To enhance the significance of the overlap, we conducted Permutation tests to obtain the unilateral *p* value for the mean PLS-Z scores of the shared genes in each disease.

### Transcriptional enrichment analysis of PLS weighted genes

To assign PLS weighted genes to specific cell types, cortical layers, and developmental stages, we performed a comprehensive multi-angle integrated enrichment analysis using multiple methods. Initially, we utilized the Cell-type Specific Expression Analysis (CSEA) (http://genetics.wustl.edu/jdlab/csea-tool-2/) to preliminarily investigate specific cell types associated with PLS1+/− genes (Dougherty, Schmidt, Nakajima, & Heintz, 2010). Additionally, we further assessed developmental time-windows across different brain regions using the CSEA tool to gain insights into the genes' relationships with developmental stages. Furthermore, to explore the potential connections between cortical layers and abnormal gradient patterns, we employed the gene set enrichment analysis (GSEA) using the ‘clusterProfiler’ and ‘GSEABase’ packages (Hung, Yang, Hu, Weng, & DeLisi, 2012). Marker datasets of six cortical layers were obtained from a previous transcriptomic study (He et al., 2017). To further validate the cellular specificity in relation to regional gradient changes, we cross-referenced the PLS1+/− genes with marker-genes of seven cortical cells, including endothelial cells, astrocytes, microglia, oligodendrocyte precursors (OPCs), oligodendrocytes, excitatory and inhibitory neurons (Li et al., 2021). These cellular markers were extracted from five independent single-cell studies conducted on human postmortem cortical tissues (Seidlitz et al., 2020).

### Analysis of age correlation and treatment prediction

To explore the relationship between age of onset and disease, we first investigated the correlation between onset's age and clinical symptoms. Then, all FES were then divided into two subgroups, including minor FES (less than 18 years of age at first onset) and adult FES (at least 18 years of age at first onset). We compared gradient metrics between two subgroups by using a GLM, with age and gender as covariates. Two-sided *t* tests (contrast = FES-HC) were performed. The global gradient analysis was corrected using the FDR correction of *p* < 0.05, and the voxel-based gradient analysis was corrected using the GRF correction (voxel *p* < 0.001, cluster *p* < 0.05).

Next, paired *t* tests were used for compare the differences in clinical symptoms of FES before and after treatment, including PANSS positive, negative, and total scores. Furthermore, the SVR was utilized to assess the predictive capability of connectome gradients concerning treatment outcomes in FES (Ouyang et al., 2020). The gradient maps at baseline were utilized as predictive features, with the model's validity confirmed through the leave-one-out cross-validation and Permutation tests. A comprehensive elucidation of the SVR model was provided in the online Supplementary material**.**

### Reproducibility verification

To ensure the reliability and robustness of our findings, we conducted a series of verification analyses. Firstly, we categorized all participants into minor and adult subgroups, subsequently evaluating the case–control *z* maps in the functional gradient for both age subgroups separately. Secondly, we assessed the stability of the case–control *z* maps in the functional gradient after the adjustment of FD. Finally, we randomly divided FES patients into two subgroups to compare the difference of case–control *z* maps in the functional gradient with HC.

### Null model

To mitigate potential confounding effects arising from spatial autocorrelation, we employed a null model utilizing the spin test (Váša et al., 2018). This method generates a series of null Spearman correlation coefficients through the spherical projection of spatial maps that have been randomly rotated, preserving their spatial relationships. In our study, we established a null distribution by performing 10 000 spin permutations tests on cortical regions. The *p*_spin_ value was subsequently calculated by determining the proportion of instances in which the null correlation coefficients exceeded the observed values.

## Results

### Demographic characteristics

**Figure 1.**
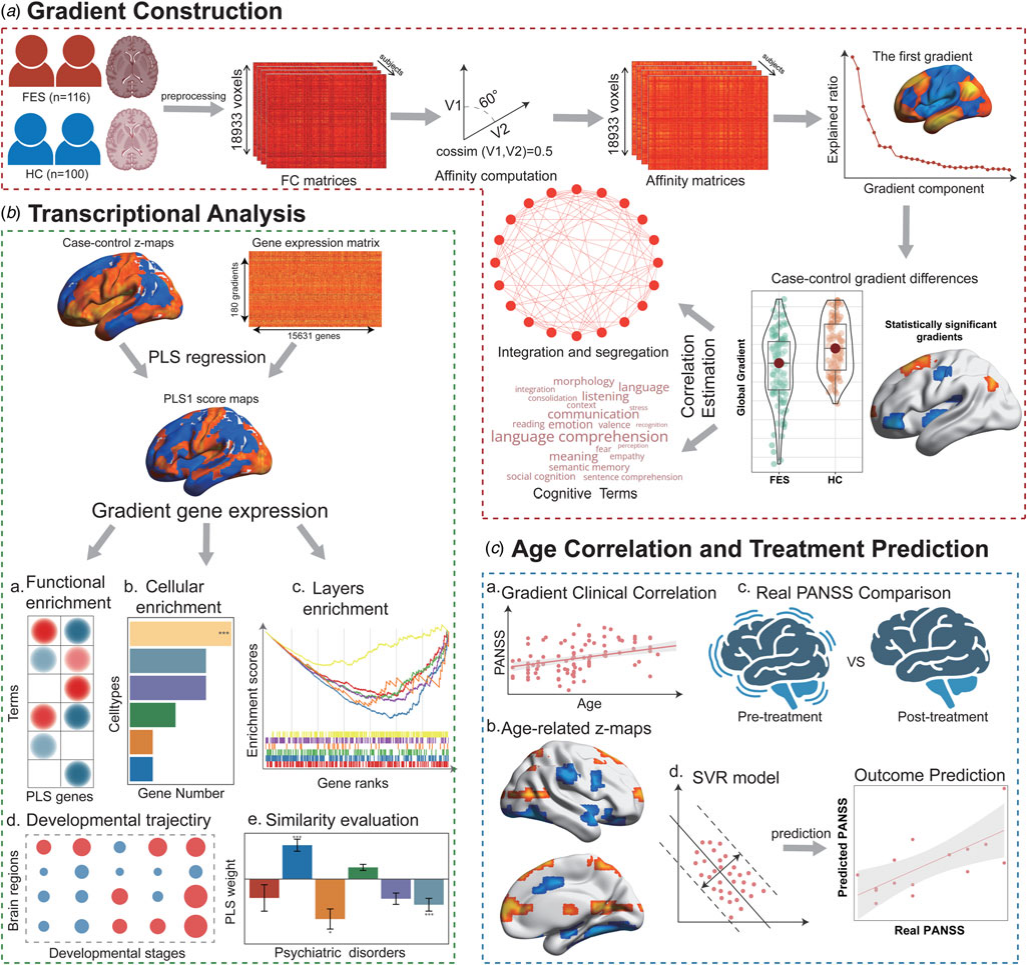
Methodological Overview. (a) Gradient construction. Firstly, the voxel-based functional connectivity gradient was calculated. The first gradient was subsequently employed to identify disparities between FES and HC. Additionally, the *z* value map was utilized to ascertain correlations with network topology and cognitive terms. (b) Transcriptional analysis. Using the AHBA database, PLS regression was harnessed to discern imaging-transcriptomic associations in FES. The relationship between case–control *z* maps and gene expression data was probed via functional enrichment of PLS weighted genes, shared genetic predispositions with other psychiatric disorders, and the transcriptional signature appraisal of cell types, cortical layers, and developmental phases. (c) Age correlation and treatment prediction. We evaluated the relationship between age of onset and the clinical manifestations of FES. Then, we compared the gradient maps of FES with an onset age of minor FES (less than 18 years of age at first onset) and adult FES (at least 18 years of age at first onset). Furthermore, PANSS scores of FES were juxtaposed pre- and post-treatment. Moreover, the gradient maps of FES at baseline were employed to forecast treatment outcomes utilizing the SVR model.

This study investigated functional gradient deficits in drug-naïve FES and examined their associations with specific transcriptional expression profiles and treatment outcomes (Fig. 1). The demographic characteristics, including sex, age, and FD, displayed no statistically significant differences between FES (mean age: 16.62 ± 3.71) and HC (mean age: 17.28 ± 4.33) (online Supplementary Table S1). Furthermore, the age distribution within FES and HC was illustrated in the online Supplementary Fig. S1.

### Functional gradient alterations in FES

**Figure 2.**
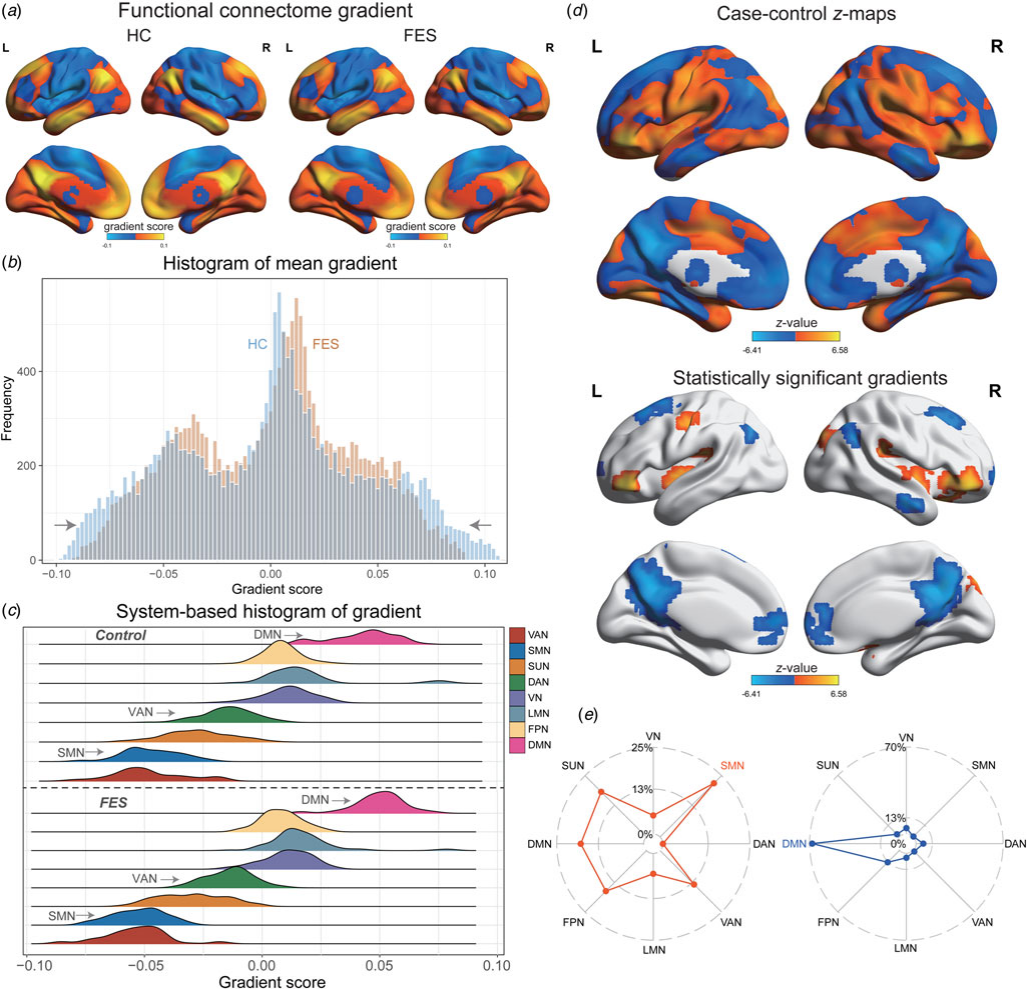
Comparison of functional gradient between FES and HC. (a) Functional gradient mapping in both FES and HC; (b) Voxel-based distribution of mean gradients; (c) Distribution of subnetwork-based functional gradients; (d) Case–control *z* map showcasing differences in functional gradients between FES and HC; (e) The radar chart illustrates the proportion of each subnetwork within distinct brain regions.

The first gradient delineated the predominant primary-to-transmodal continuum, accounting for 12.4% of the total connection gradient's variance (FES: 11.7%; HC: 13.3%; online Supplementary Fig. S2), which was systematically arranged from the primary visual/sensorimotor networks (VN/SMN) towards the DMN (Fig. 2a). Remarkably, the spatial correlations of the group-averaged principal gradient maps exhibited substantial similarity between FES and HC (online Supplementary Fig. S3). A detailed examination of the histogram indicated a contraction in the extremes of the primary-to-transmodal gradient for FES compared to the HC (Fig. 2b). In terms of subnetworks, we juxtaposed the gradient of the group-averaged maps between FES and HC, employing two-sample *t* tests and controlling for gender and age (FDR-corrected *p* < 0.05). The FES demonstrated increased gradient in the SMN (Cohen's *d* = 0.45, *p* = 0.004) and ventral attention network (VAN; Cohen's *d* = 0.36, *p* = 0.023), and a decrease in the DMN (Cohen's *d* = −0.53, *p* = 0.001; Fig. 2c; online Supplementary Table S2).

**Figure 3.**
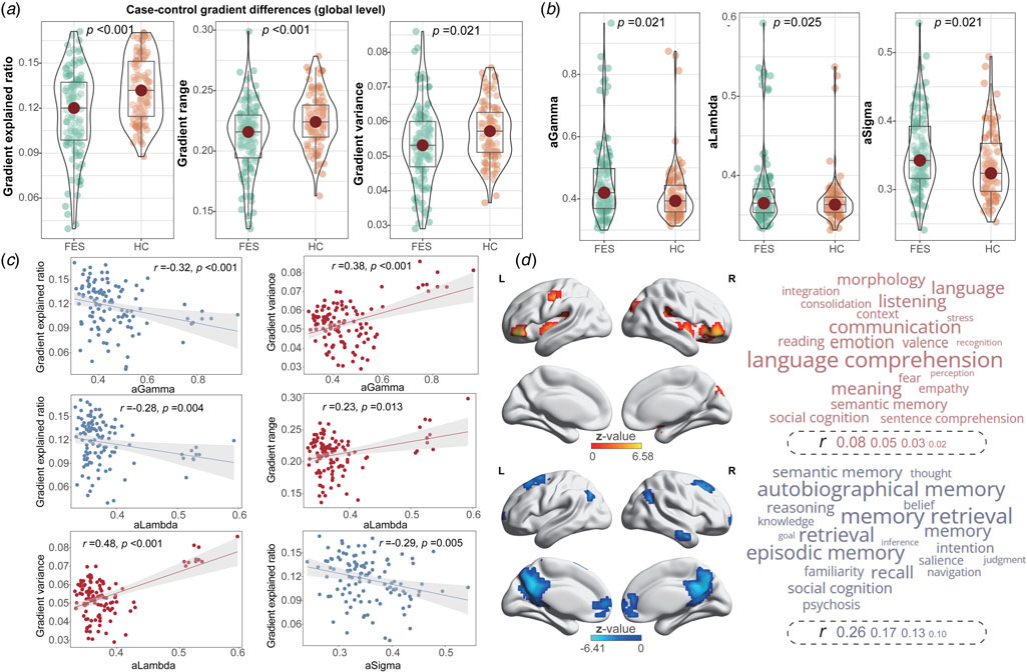
Statistical comparisons of gradient metrics. (a) Global gradient differences between FES and HC; (b) Network topology differences between FES and HC; (c) Correlations between global gradients and network topology among FES; (d) Word clouds representations of cognitive terms linked to case–control *z* map for FES.

Regionally, FES displayed decreased gradients in the middle frontal gyrus, angular gyrus, right middle temporal gyrus, anterior cingulate gyrus, left cuneus, and left medial superior frontal gyrus. Conversely, FES exhibited increased gradients in the left postcentral gyrus, putamen, inferior orbitofrontal gyrus, and right cuneus (Fig. 2d; online Supplementary Table S3). These alterations were primarily characterized by decreased gradients within the DMN (67.6%) and increased gradients in the SMN (21.9%; Fig. 3e). Globally, FES had a decreased explained ratio (Cohen's *d* = −0.57, *p* < 0.001), a narrower gradient range (Cohen's *d* = −0.49, *p* < 0.001), and diminished gradient variance (Cohen's *d* = −0.32, *p* = 0.021; Fig. 3a; online Supplementary Table S4). Additionally, both global and local gradient measures did not reveal a significant sex-group interaction effect.

### Correlations between network topology and global gradients in FES

For network topology, FES displayed heightened values for aGamma (Cohen's *d* = 0.36, *p* = 0.021), aLambda (Cohen's *d* = 0.31, *p* = 0.025), and aSigma (Cohen's *d* = 0.34, *p* = 0.021; Fig. 3a; online Supplementary Table S4) compared to HC, indicating that the segregation and integration functions of the onset stage in FES might be compensating to maintain efficient communication. Notably, aGamma was negatively correlated with the gradient explained ratio (*r* = −0.32, *p* < 0.001) and positively with the gradient variance (*r* = 0.38, *p* < 0.001) in FES. aLambda was negatively correlated with the gradient explained ratio (*r* = −0.28, *p* = 0.004) while positively correlated with both the gradient range (*r* = 0.23, *p* = 0.013) and the gradient variance (*r* = 0.48, *p* < 0.001). Additionally, aSigma was negatively correlated with the gradient explained ratio (*r* = −0.29 *p* = 0.005; Fig. 3c).

### Meta-analytic cognitive terms linked to gradient changes in FES

The *z* map, reflecting increased gradients in FES, exhibited correlations with meta-analytic cognitive features primarily associated with comprehension, communication, language, and emotion. However, these correlations did not withstand FDR correction. Conversely, the *z* map denoting decreased gradients in FES was linked to cognitive terms chiefly concerning memory, recall, social cognition, and thought (Fig. 3d; online Supplementary Table S5).

### Transcriptional patterns related to gradient changes in FES

**Figure 4.**
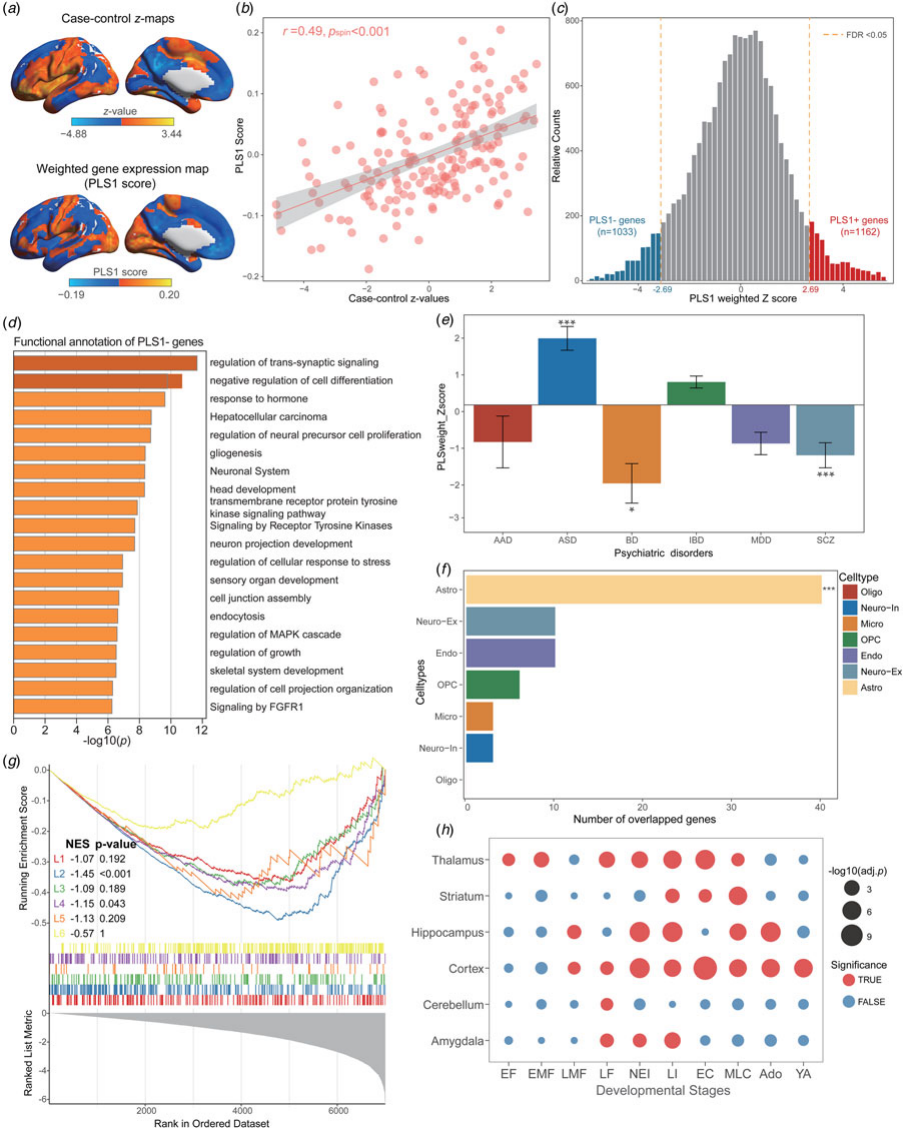
Transcriptional analysis of PLS weighted genes associated with case–control gradients. (a) The coincident distribution between case–control *z* maps of gradient changes and weighted gene expression-map of PLS1 scores in the left hemisphere. (b) The scatterplot displayed a notable positive spatial correlation between PLS1 scores and the case–control *z* value maps for FES (*r* = 0.49, *p*_spin_ < 0.001). (c) A total of 1162 PLS1+ genes (*Z* > 2.69, FDR-corrected *p* < 0.05) and 1033 PLS1− genes (*Z* < −2.69, FDR-corrected *p* < 0.05) were discerned by ranked *Z* scores. (d) The top 20 terms from functional enrichment of PLS1− genes were determined using Metascape software. (e) PLS1 weights exhibited higher correlations with dysregulated genes from ASD, BD and adult-SCZ (FDR-corrected *p*_perm_ < 0.05) by intersecting common genes, while no significant differences were detected in the dysregulated genes from MDD, IBD and AAD (FDR-corrected *p*_perm_ > 0.05). (f) The number of genes overlapping with PLS1− genes was analyzed for each cell type, and only astrocyte exhibited significant overlap as determined by Permutation tests (number = 40, FDR-corrected adjusted *p*_perm_ < 0.001). (g) The GSEA enrichment indicated that PLS1− gene list was significantly enriched in layer II (NES = −1.45, *p* < 0.001) and layer IV (NES = −1.15, *p* = 0.043). (h). The developmental gene expression enrichment analysis revealed that PLS1− genes primarily express in the brain regions from late mid-fetal to young adulthood stages, notably across cortex, striatum, thalamus, and hippocampus.

The first PLS component (PLS1) effectively accounted for the majority of gradient variations (24.1%) in FES-related changes, significantly exceeding the expectation by chance (*p*_spin_ < 0.001, online Supplementary Fig. S4). Strikingly, the distribution of the PLS1 scores exhibited higher expression primarily in the posterior parietal-occipital areas, while lower expression was observed in the prefrontal regions, aligning with the case–control *z* maps of gradient changes in FES (Fig. 4a). The regional mapping of PLS1 scores displayed significant spatially positive correlations with the case–control *z* maps of gradient abnormities (*r* = 0.49, *p*_spin_ < 0.001, Fig. 4b). Subsequently, PLS1 weighted genes were identified by a univariate one-sample *Z* test and a total of 1162 PLS1+ genes and 1033 PLS1− genes (FDR-corrected *p* < 0.05) were respectively identified according to their *Z* score ranks (Fig. 4c).Functional enrichment analysis indicated that PLS1− genes were significantly enriched in pathways linked to synaptic signaling and neural development processes, including ‘regulation of trans-synaptic signaling’, ‘negative regulation of cell differentiation’, ‘regulation of neural precursor cell proliferation’, and ‘neuronal system’ (Fig. 4d). Contrastively, PLS1+ genes were notably enriched in energy metabolism-related biological processes, such as ‘lipid biosynthetic process’, ‘membrane lipid metabolic process’ and ‘RAC1 GTPase cycle’ (online Supplementary Fig. S5a). Moreover, adult-SCZ-related and BD-related genes exhibited stronger negative PLS1 weights, while ASD-related genes displayed stronger positive PLS1 weights than that of other disorders (FDR-corrected *p*_perm_ < 0.05, Fig. 4e). These findings suggested a closer association of PLS1− genes with the transcriptional signatures of classical FES.

### Transcriptional signatures assessment for enrichment of specific cell types, cortical layers, and developmental stages in FES

To delve further into the cellular-level transcriptional signatures alongside regional gradient changes in FES, we conducted the CSEA analysis. This analysis unveiled a significant connection between the PLS1− gene-list and astrocytes, as well as neurons in different brain regions, whereas the PLS1+ genes were predominantly linked to oligodendrocyte progenitors (online Supplementary Fig. S5b, c). Moreover, to confirm the cellular specificity to gradient changes, we executed a permutation test using the overlapped genes from seven cell types. This analysis indicated a notable association between PLS1− genes and astrocytes (number = 40, FDR-corrected adjusted *p*_perm_ < 0.001, Fig. 4f), while PLS1+ gene-list was significantly related to oligodendrocytes (number = 80, FDR-corrected adjusted *p*_perm_ < 0.001, online Supplementary Fig. S5d), consistent with results of CSEA. For the cortical layer enrichment analysis, the PLS1− gene-list displayed significant enrichments in layer II (NES = −1.45, *p* < 0.001) and layer IV (NES = −1.15, *p* = 0.043, Fig. 4g), while the PLS1+ genes exhibited no significant enrichments across cortical layers (*p* > 0.05, online Supplementary Fig. S5e). Interestingly, the developmental enrichment analysis indicated that PLS1− genes exhibited predominant expression in the brain regions from late/mid fetal (LMF) to young adulthood (YA) stages, particularly across cortex, striatum, thalamus, and hippocampus (Fig. 4h). In contrast, the PLS1+ genes were significantly enriched in stages from early childhood (EC) to YA, and exerted a prominent impact on regions such as striatum, hippocampus and amygdala (online Supplementary Fig. S5f).

### Age correlation and treatment prediction in FES

**Figure 5.**
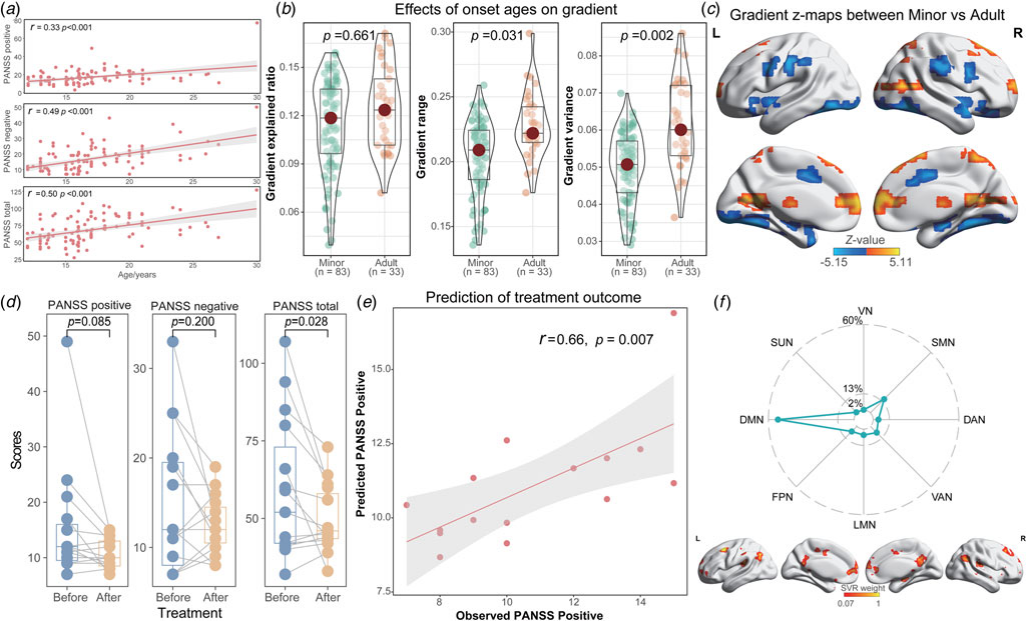
Analysis of age correlation and treatment prediction. (a) Correlation between age of onset and clinical symptoms among FES; Differences of global (b) and voxel-based (c) gradients between minor FES (less than 18 years of age at first onset) and adult FES (at least 18 years of age at first onset); (d) Variations in clinical symptoms in FES pre- and post-treatment; (e) Treatment prediction using SVR model; (f) Absolute sum of weights derived from subnetworks; The radar chart depicts the distribution of predictive power across various systems.

The onset age of FES exhibited positive correlations with PANSS positive (*r* = 0.33, *p* < 0.001), negative (*r* = 0.49, *p* < 0.001), and total scores (*r* = 0.50, *p* < 0.001; Fig. 5a), implying that FES may manifest milder clinical symptoms in younger stages. Compared to adult FES, minor FES exhibited reduced gradient range (Cohen's *d* = −0.45, *p* = 0.047) and gradient variance (Cohen's *d* = −0.66, *p* = 0.006; Fig. 5b). Furthermore, voxel-based gradient analysis revealed elevated gradients in the minor FES group predominantly in the superior orbitofrontal gyrus, inferior parietal gyrus, middle temporal gyrus, and hippocampus, whereas decreased gradients were observed mainly in the caudate nucleus, medial superior frontal gyrus, and left precuneus (Fig. 5c; online Supplementary Table S6).

Additionally, FES displayed a reduction of clinical symptoms in PANSS total scores (Cohen's *d* = 0.63, *p* = 0.028) and positive scores (Cohen's *d* = 0.48, *p* = 0.085) following 8-week treatment. Importantly, gradient maps of FES at baseline could significantly forecast the post-treatment PANSS positive scores (*r* = 0.66, *p* = 0.007; mean square error (MSE) = 3.91, *p*_perm_ = 0.017; Fig. 5e). The most influential features contributing to this prediction were predominantly located within the DMN (53.6%) and SMN (15.1%), representing the proportion of total feature weights in SVR (Fig. 5f).

### Reproducibility analysis of functional connectivity gradient

Considering the broad age range in this study, we categorized all participants into minor and adult groups. The case–control *z* maps for FES patients and HC were then analyzed separately for minors and adults **(**online Supplementary Fig. S6). The *z* maps across all participants exhibited spatially positive correlations with both the minor group (*r* = 0.88, *p*_spin_ < 0.001) and the adult group (*r* = 0.74, *p*_spin_ < 0.001). Subsequently, we explored the impact of FD on the observed case–control differences in functional gradient (online Supplementary Fig. S7). Notably, the *z* maps of functional gradient between FES and HC, controlling for FD, remained highly correlated with those unadjusted for FD (r = 0.99, *p*_spin_ < 0.001). Finally, to evaluate potential data heterogeneity, we randomly divided the FES patients into two subgroups and compared their functional gradient with that of HC (online Supplementary Fig. S8). The *z* maps for FES patients across all participants exhibited significant spatially positive correlations with both subgroup1 (*r* = 0.97, *p*_spin_ < 0.001) and subgroup2 (*r* = 0.96, *p*_spin_ < 0.001).

## Discussion

Our study represents the initial comprehensive exploration of complex interplay between voxel-based functional gradients and underlying molecular mechanisms in drug-naïve FES, by assessing the relationship between abnormal functional gradients, specific transcriptional expression patterns, and treatment outcomes. Our findings delineated abnormal functional connectivity gradient patterns in FES, with disrupted functional integration and separation mechanisms, as well as cognitive dysfunction. Moreover, our investigation also revealed a significant overlap of genes between PLS1 weighted genes linked to gradient alterations of FES and the dysregulated genes reported in adult SCZ, BD, and ASD. Of particular note, genes correlated with gradients changes exhibited a significant enrichment not only in signaling pathways and biological processes, but also in specific cell types, cortical layers, and developmental stages. Furthermore, we identified a positive association between the severity of FES and the age of first onset, and distinct functional gradient patterns between minor FES and adult FES. Additionally, the connection gradient in FES at baseline could predict positive symptoms after treatment. These findings provide a novel perspective for the intricate relationship between macroscopic functional connectivity gradient alterations and microscopic transcriptional patterns during the onset and progression of FES.

Voxel-based functional connectivity gradient analysis unveiled abnormalities in the principal primary-to-transmodal gradient pattern among FES (Holmes et al., 2023). Our results indicated a reduced explanation ratio of the principal gradient, suggesting decreased variance in functional connectome connectivity in FES. Furthermore, the diminished range and variance hinted at less distinct connectivity patterns between primary and transmodal areas in FES. Notably, the most pronounced differences in FES were observed in SMN and DMN, situated at the two extremes of the principal gradient (Hu et al., 2017). The SMN is pivotal in the specialized processing of sensory stimuli and motor responses (Chenji et al., 2016), while the DMN is primarily involved in internally directed processes, including conceptual processing, self-monitoring, and both autobiographical and spontaneous cognition (Yeshurun, Nguyen, & Hasson, 2021). Gradient anomalies in functional networks suggested that FES exhibited abnormal cortical hierarchical organization and information conversion disorders in tasks involving sensorimotor and ruminative thinking (Conio et al., 2020). Furthermore, deviations in network topology were correlated with anomalous connectivity gradients, indicating that FES were compensating through altered patterns of segregation and integration, which were consistent with the principal gradient's deficit (Ouyang, Kang, Detre, Roberts, & Huang, 2017). Our results establish a connection between network gradients and topological alterations in FES.

Additionally, our meta-analysis ascertained that regions primarily displaying decreased connectivity gradients in FES were significantly associated with cognitive terms, which encompass various higher cognitive functions such as memory, recall, social cognition, and thought processing (Wei et al., 2023). These findings offer a potential biological explanation at a physiological level for the relationship between disrupted connectome gradients and cognitive deficits in FES. Furthermore, the age at first onset influences the progression of the disease (Jeremian et al., 2022). The relationship between onset age and variations in clinical symptoms remains controversial. Prior studies have indicated that EOS was associated with more pronounced negative symptoms, whereas late-onset SCZ was characterized by more pronounced positive symptoms (Sato, Bottlender, Schröter, & Möller, 2004). However, in our study, age-related analyses revealed that FES with an earlier onset exhibited milder positive and negative symptoms, suggesting that the impact of onset age on symptom patterns might differ between FES and adult-onset SCZ. Notably, younger FES displayed distinct connection gradient patterns compared to adult FES, indicating that varying ages at first onset correspond to specific patterns of cortical hierarchy organization (Heinz et al., 2019). Moreover, the connection gradients in FES at baseline robustly predicted positive symptoms after treatment. The DMN plays a pivotal role in our predictive model for treatment outcomes. Our findings suggest that the DMN connectivity and its distinctive patterns of interaction with other systems may serve as potential indicators of clinical outcomes in FES.

Based on PLS1 weighted genes, the functional enrichment analysis shed light on the transcriptional signatures pertaining to functional gradient abnormities in FES. Intriguingly, PLS1− genes displayed substantial enrichments in pathways linked to synaptic signaling and neural development processes. Synaptic signaling, crucial for maintaining synaptic stability and maturation, is intricately connected to the mechanisms underlying SCZ via regulating neuron activity, connectivity, and co-transmission (Chuhma, Mingote, Kalmbach, Yetnikoff, & Rayport, 2017). Conversely, PLS1+ weighted genes were notably enriched in biological processes linked to energy metabolism. Dysregulation of various metabolites, including lipids, amino acids, and sulfur compounds, has been reported to be associated with the multifaceted pathogenesis of SCZ, influencing cellular homeostasis and tissue integrity (Chen et al., 2020; Palego, Betti, & Giannaccini, 2015). Previous GWAS studies have emphasized the existence of shared genetic foundations between SCZ and BD. These shared genes, including *CDH13*, *AMBRA1*, *ARNTL*, *EFHD1*, *PLXNA2*, *ANK3*, *UGT1A1*, and *MHC*, are implicated in synaptic plasticity, neurodevelopment, immune system function, and neurocognitive impairment (Prata, Costa-Neves, Cosme, & Vassos, 2019). Additionally, Liu et al., successfully identified synaptic activity and immunity as the shared mechanisms in ASD and SCZ using next-generation sequencing technologies (Liu, Li, Fan, Zhang, & Chen, 2017). In alignment with these forementioned studies, our results revealed a significant overlap of genes between PLS1 weighted genes links to gradient differences, and the dysregulated genes reported in adult-SCZ, BD, and ASD, confirming the shared genetic commonalities and similar psychopathological abnormities among these psychiatric disorders.

Cellular irregularities and the positioning of cortical neurons are pivotal in the development of psychiatric disorders, encompassing ASD, BD, MDD, and SCZ. Using the specific biomarkers of distinct cell types and cortical layers, our study unveiled notable enrichments of PLS1− genes in astrocytes, while PLS1+ genes in oligodendrocytes. Astrocytes are recognized as essential contributors to crucial neurodevelopmental and homeostatic processes, integral to the pathogenesis of SCZ through their involvement in synaptogenesis, glutamatergic signaling, and myelination (Notter, 2021). Oligodendrocyte deficits have been demonstrated associated with impaired maturation and disrupted regeneration, contributing to cognitive deficits in SCZ (Raabe et al., 2019). Additionally, our investigation unveiled that PLS1− genes were primarily enriched in specific cortical layers, particularly in layer II and IV, while no significant layer-related enrichments were identified for PLS1+ genes. This observation suggests that the distinctive distribution of diverse neurons and their connections within affected regions may offer insights into the underlying nature of potential pathology in FES (Batiuk et al., 2022). Furthermore, our developmental enrichment analysis indicated that the PLS1− genes were predominantly expressed in regions from LMF to YA stages, particularly across cortex, striatum, thalamus, and hippocampus. In contrast, the PLS1+ genes were significantly enriched in stages from EC to YA, exerting a prominent influence on regions such as striatum, hippocampus and amygdala. This reveals that susceptibility to FES may occur within distinct potential time windows, contingent upon varying transcriptional irregularities aligned with corresponding functional gradient changes (Talarico et al., 2022).

However, several limitations still exist in this study. Firstly, the sample size of this study is relatively limited, and more participants need to be collected in the future to further verify our conclusions. Secondly, although we explored potential associations between the gradient changes and cognitive abnormities using the meta-analytic cognitive terms, the lack of real clinical cognitive datasets remains a limitation. As such, the brain-cognition correlations in FES need further estimation and validation through additional clinical retrospective studies. Thirdly, the transcriptional datasets, obtained from the AHBA database, only cover limited gene expression profiles from two right hemisphere. Hence, we only selected the transcriptional signatures of left hemisphere in subsequent analysis and there still lack corresponding connection between gene signatures and functional gradient changes in the right hemisphere. Finally, this study only included a limited set of patients who received standard treatment and had an 8-week period to estimate the predictive capability of functional gradient abnormities on treatment outcomes. Future research should aim to enlarge the cohort of patients undergoing treatment and prolong the duration of follow-up.

## Conclusion

This study provided an exhaustive analysis of functional connectivity gradient abnormalities in FES, revealing widespread anomalies in the SMN and DMN. These abnormalities were intricately linked to both compensatory mechanisms of integration and segregation and to deficits in higher-order cognitive functions. Furthermore, genes associated with these gradient abnormalities were enriched in pathways related to synaptic signaling and neurodevelopment, and showed associations with other psychiatric disorders, specific cell types, and cortical layers. Additionally, the age of onset influenced the severity observed in FES, resulting in distinct connection gradient patterns between minor and adult FES. Moreover, the connectivity gradients of FES at baseline could predict treatment outcomes. These findings lay a crucial theoretical groundwork for understanding the complex relationship between macroscopic functional connection gradient changes and microscopic transcriptional patterns during the onset and progression of FES.

## Supporting information

Yao et al. supplementary material Yao et al. supplementary material

## Data Availability

The code for calculating the gradient could be found on the github: https://github.com/mingruixia/MDD_ConnectomeGradient. The code for PLS analysis could be available at the github: https://github.com/SarahMorgan/Morphometric_Similarity_SZ. The code for the computation of spatial permutation testing could be available at the github: https://github.com/frantisekvasa/rotate_parcellation. The gene expression datasets could be available at the AHBA database: http://human.brain-map.org. The datasets of meta-analytic cognitive terms could be available at the website: https://neurosynth.org/. Other data will be made available upon request.

## Supplementary material

The supplementary material for this article can be found at https://doi.org/10.1017/S0033291724001739
