# Supplementary material for: Functional gradient dysfunction in drug-naïve first-episode schizophrenia and its correlation with specific transcriptional patterns and treatment predictions
Source: Psychol Med. 2024 Nov 18;54(15):4106–18. doi: 10.1017/S0033291724001739 (PMC11650189; doi:10.1017/S0033291724001739)
Supplement: Yao et al. supplementary material [file S0033291724001739sup001.docx]

**Supplementary Methods:**

**Imaging acquisition and preprocessing**

Imaging data were acquired using a 3-Tesla MRI scanner (MAGNETOM Verio; Siemens, Germany) at the First Hospital of Shanxi Medical University. Resting-state functional MRI (rs-fMRI) scans employed a two-dimensional echo-planar imaging (EPI) sequence with the following parameters: TR = 2,500 ms, TE = 30 ms, flip angle = 90°, matrix = 64 × 64, 212 volumes, slice thickness of 3 mm with a 1 mm gap, and a voxel size of 3.75 × 3.75 × 4 mm^3^ across 32 axial slices.

The rs-fMRI images were preprocessed using SPM12 (www.fil.ion.ucl.ac.uk/spm/) and DPARSF (http://www.restfmri.net). The initial ten time points were discarded for stability. Preprocessing incorporated slice-timing correction and motion correction. Participants exhibiting translations exceeding 2.5 mm or rotations greater than 2.5° were excluded to reduce the impact of head movements. Additionally, frame displacements (FD) were computed, and frames with displacements surpassing 0.50 were omitted. The functional images were subsequently normalized to standard space using an echo-planar imaging (EPI) template, resampled to 3-mm isotropic voxels, and then smoothed employing a 6-mm full-width at half-maximum Gaussian kernel. Linear detrending was conducted, and potential confounds such as the Friston-24 motion parameters, white matter, and cerebrospinal fluid signals were regressed from the voxel time series. Temporal bandpass filtering within the range 0.01-0.08 Hz was then executed.

**Network topology construction**

The shortest path length denotes the mean distance between two nodes in a network (Leming, Su, Chattopadhyay, & Suckling, 2019). Lambda represents the ratio of this length in the small-world network to that of a random network. The clustering coefficient quantifies the connectivity among a node’s neighbors (Slinger, Otte, Braun, & van Diessen, 2022). Gamma is the ratio of the clustering coefficient of the small-world network to that of the random network. The small-world network (Sigma) is characterized by the ratio of Gamma to Lambda, reflecting a balance between integration and segregation functions (Liao, Vasilakos, & He, 2017).

For each imaging dataset, a 360×360 symmetric matrix was derived by calculating Pearson correlation coefficients on a region-to-region basis (Glasser et al., 2016). To improve data normality, matrices underwent Fisher’s-*z* transformation. Weighted matrices were then generated based on the matrix threshold. Network metrics were assessed using the GRETNA toolbox (https://www.nitrc.org/projects/gretna/). The negative correlation was set to zero, consistent with previous studies on the construction of functional connectivity networks (Narr & Leaver, 2015). Network properties were evaluated within the sparsity (S) threshold range of 0.10-0.40, incrementing by 0.01. The area under the curve (AUC) was computed for each network metric, providing a holistic measure to quantify the topological attributes of brain networks.

**Association of meta-analytic cognitive terms with gradient alterations in FES**

The Neurosynth eliminated noncognitive terms, retaining 123 cognitive terms (Hansen et al., 2021). To assess the significance of each cognitive term’s correlation coefficients, we employed Permutation tests (10,000 iterations), ensuring the correction of spatial autocorrelations through generative modeling. For each iteration, the between-group difference *z*-map of the connectome gradient underwent a randomized voxel shuffle. Subsequently, the original *z*-map’s variogram was calculated to smooth and recalibrate the permuted map, yielding a spatial autocorrelation-preserving surrogate *z*-map. These surrogate *z*-maps were subjected to a threshold (voxel-level *p* <0.001 and cluster-level GRF-corrected *p* <0.05) and categorized into FES-positive and FES-negative maps. We then calculated the spatial correlations between the meta-analytic map for each cognitive term and the surrogate map. After 10,000 iterations, a null model was constructed. The real correlation coefficient’s *r*-value for each cognitive term was gauged against its position in the null model, with the FDR applied for multiple comparison corrections. Lastly, the top 20 cognitive terms related to FES-related gradient changes were displayed in a word-cloud plot, with font size reflecting their correlation strength with the corresponding meta-analytic maps from Neurosynth.

**SVR model**

We conducted the SVR (www.csie.ntu.edu.tw/~cjlin/libsvm/) using a linear kernel to assess the predictive capability of the gradient maps to predict the symptom scores of patients after treatment. We employed the leave-one-out cross-validation (LOOCV) technique to gauge the predictive accuracy, using data from one patient as the test set and the rest as the training set. To counteract potential bias from features with large numerical ranges, we implemented *z*-normalization for each feature in the training dataset and applied these normalization parameters to the test dataset. Subsequently, feature screening was executed, and voxels with a correlation *p*-value less than 0.005 between the training set’s gradient map and symptom score were selected as features. The accuracy was denoted by the Spearman’s correlation coefficient comparing predicted and actual symptom scores across patients.

A permutation test, using 1,000 permutations, was conducted by randomly redistributing the observed clinical scores among participants to ascertain if the observed correlation was merely coincidental. Each permutation involved a full replication of the SVR process, inclusive of feature selection. Furthermore, the significance of each voxel-based functional gradient within the SVR model was quantified by calculating its weight score. This score was determined by aggregating the instances where the feature was non-zero across all folds. If a feature was not chosen in a particular fold, its contribution to that fold was designated as zero.

**Supplementary Figures:**

**
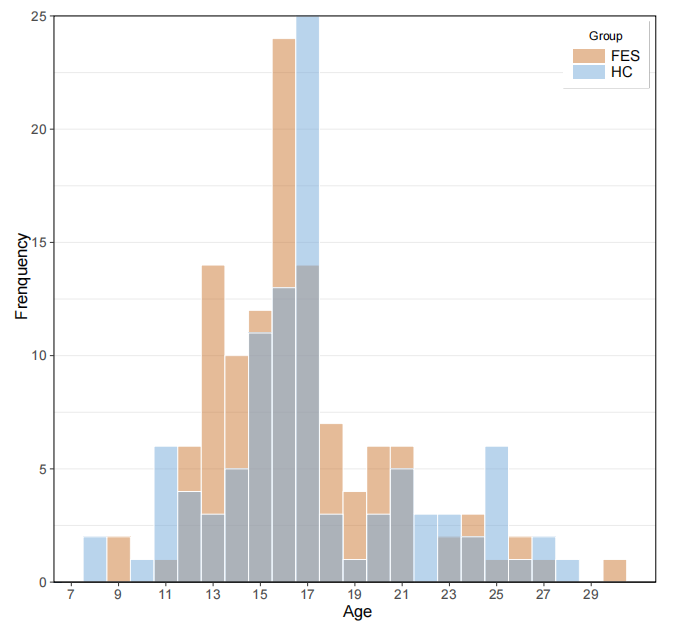
**

**Figure. S1 Frequency distribution of age.** The frequency chart indicates that the age range of all participants spanned from 7 to 30 years.

**
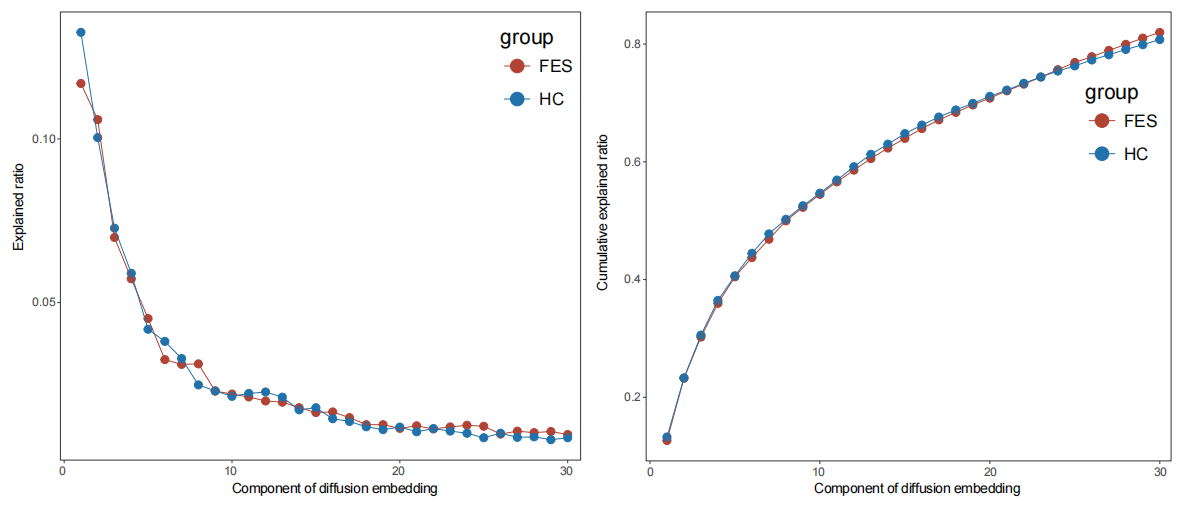
**

**Figure. S2 Explained ratio of functional connectivity gradients. (a)** The averaged explained ratio and **(b)** the cumulative averaged explained ratio of the first 30 diffusion embedding components in the FES and HC groups.

**
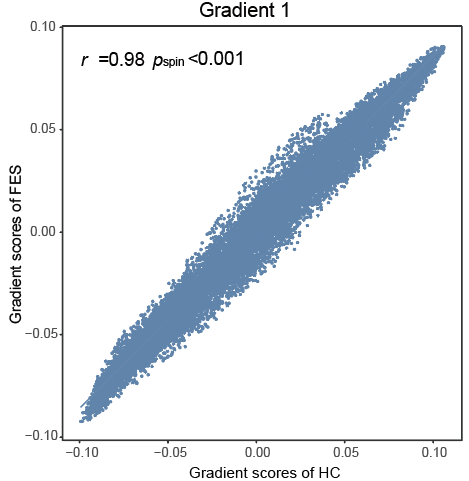
**

**Figure. S3 Spatial correlations of the group-averaged gradient maps between the FES and HC groups.** The spatial patterns of the group-averaged gradient maps were remarkably similar between the FES and HC groups (r =0.98, *p* <0.001). These correlations were corrected for spatial autocorrelations by using a permutation test (N = 10,000).


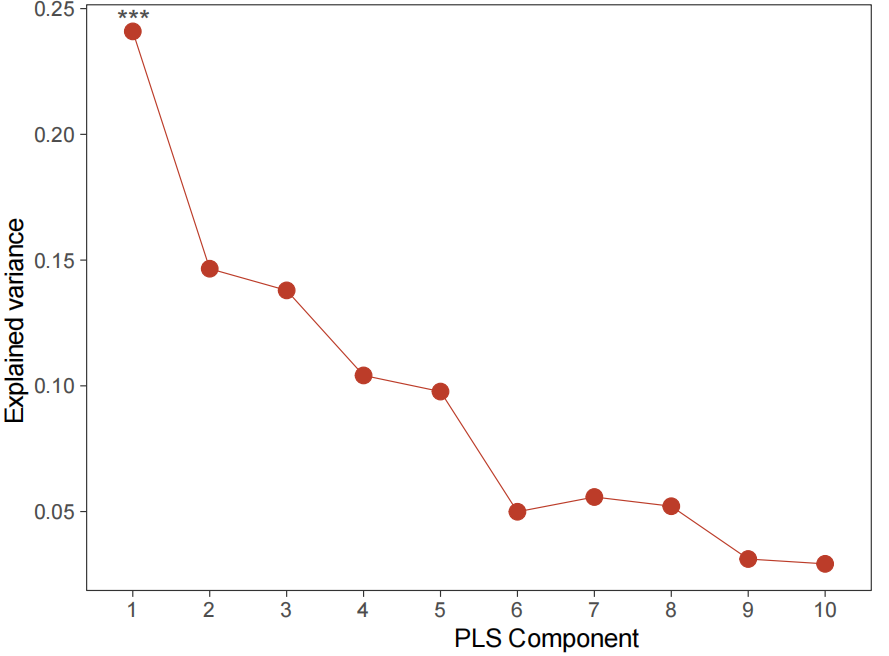


**Figure. S4** **The percentage of variance in the response variables explained by the components in the PLS analysis.** The significance level was determined by a permutation test (N = 10,000) with spatial autocorrelation corrected. ***, *p* < 0.001; PLS, partial least squares regression.

**
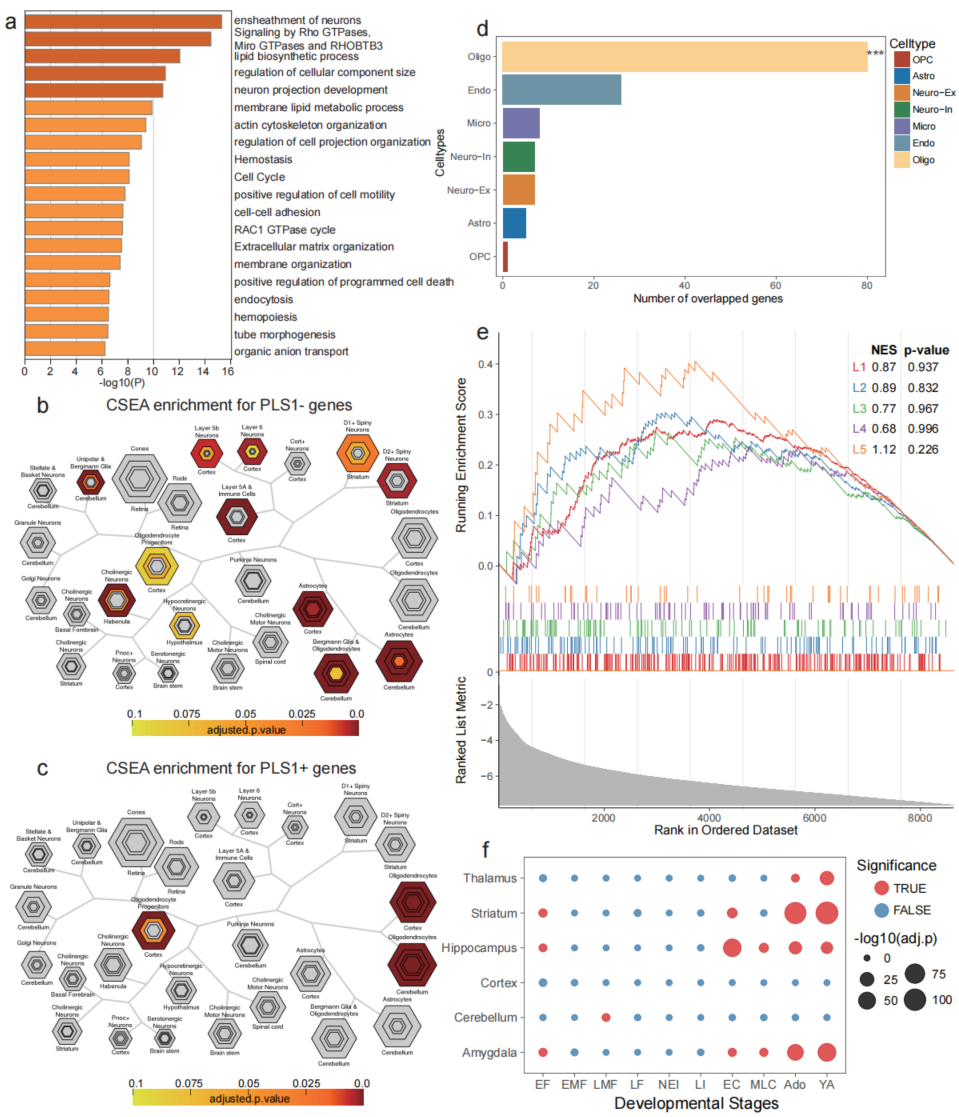
**

**Figure. S5 Transcriptional enrichment analysis of PLS weighted genes.** **(a)** The functional annotation of PLS1+ genes by Metascape. **(b-c)** The CSEA analysis revealed a significant association between the PLS1- gene-list and astrocytes as well as PLS1+ genes and oligodendrocytes. The color represents adjusted p values. **(d)** The number of overlapped genes with PLS1+ genes for each cell type and only oligodendrocyte was corrected by Permutation tests (number =80, FDR-corrected adjusted *p*_perm_ <0.001). **(e)** The GSEA enrichment shows PLS1+ genes were not enriched in any layers. **(f)** Developmental gene expression enrichment analysis showed that the PLS1+ genes exhibited predominant expression in the brain regions from EC to YA stages.

**
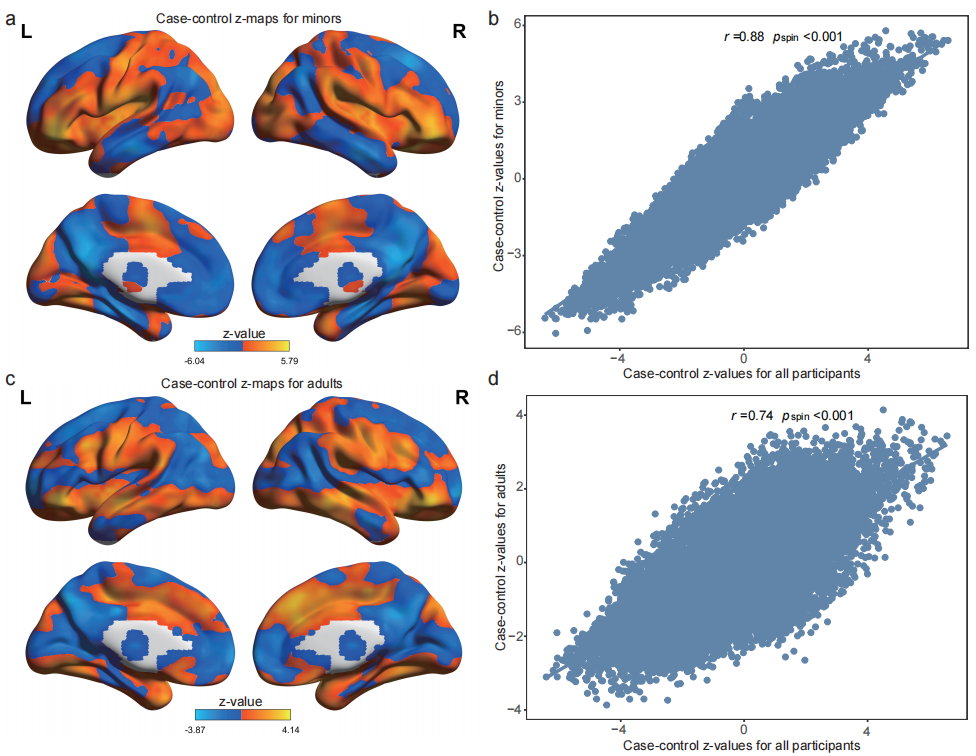
**

**Figure. S6 Spatial correlation analysis of case-control z-maps between different stages.** The case-control z-maps in minors **(a)** and adults **(c)**. The z-maps across all participants exhibited positive spatial correlations with both the minor group (r =0.88, *p*_spin_ <0.001, **b**) and the adult group (r =0.74, *p*_spin_ <0.001, **d**).


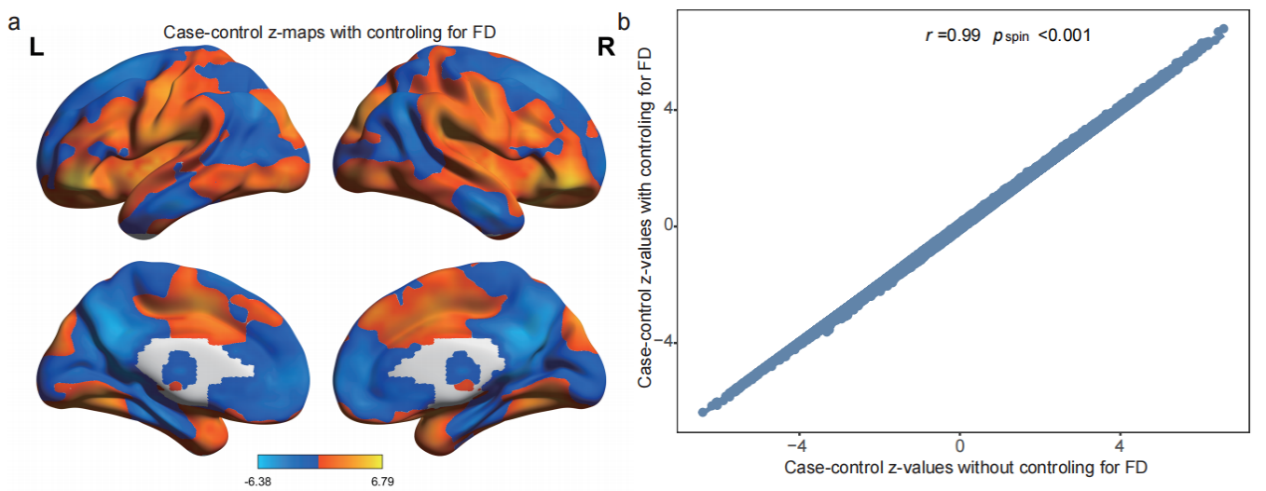


**Figure. S7 Validation of FD effect on case-control differences in functional gradient. (a)** Case-control z-maps after controlling for FD. **(b)** the z-maps of functional gradient between FES and HC, controlling for FD, remained highly correlated with those unadjusted for FD (r =0.99, *p*_spin_ <0.001).

**
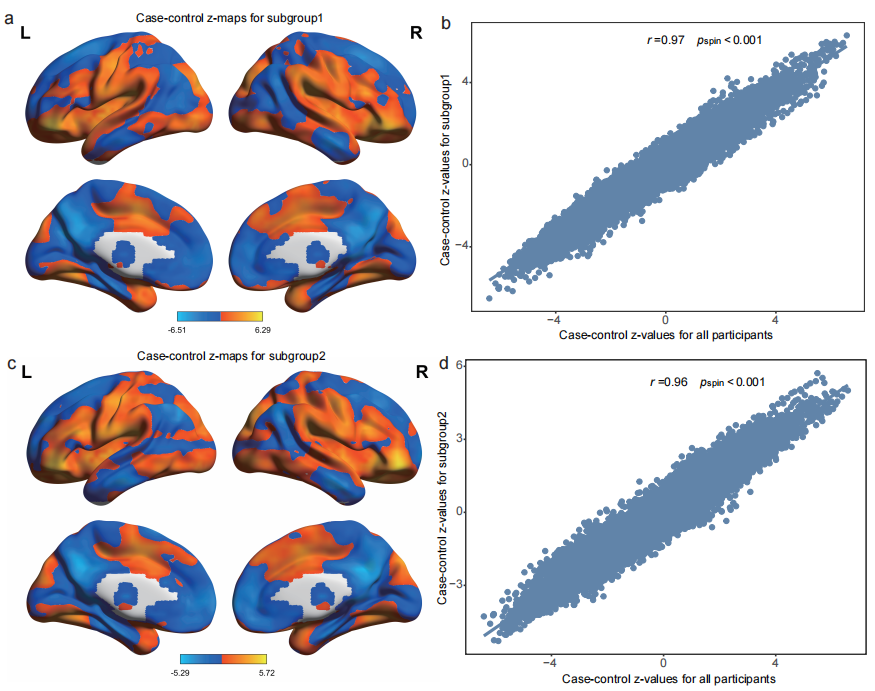
**

**Figure. S8 Spatial correlation analysis of case-control z-maps between different subgroups.** The case-control z-maps in subgroup1 **(a)** and subgroup2 **(c)**. The z-maps for FES patients across all participants exhibited significant positive spatial correlations with both subgroup1 (r =0.97, *p*_spin_ <0.001, **b**) and subgroup2 (r =0.96, *p*_spin_ <0.001, **d**).

**Supplementary Tables:**

**Table. S1** **Demographic and clinical characteristics**

| Variable | FES (n=116) | HC (n=100) | *p* |
| --- | --- | --- | --- |
| Age (year) | 16.62±3.71 | 17.25±4.33 | 0.160^a^ |
| Sex (M/F) | 50/66 | 32/68 | 0.122^b^ |
| FD | 0.07±0.04 | 0.07±0.03 | 0.860^a^ |
| PANSS positive | 17.17±8.79 |  |  |
| PANSS negative | 16.80±8.07 |  |  |
| PANSS total | 67.99±19.22 |  |  |

**Note:** FES, first-episode schizophrenia; HC, healthy controls; FD, framewise displacement; M/F, male/female; PANSS, Positive and Negative Symptom Scale; ^a^Mann-Whitney *U*-test (two-sided); ^b^Fisher’s exact test.

**Table. S2 Between-group differences in the gradient scores of the subnetworks in the mean maps**

| Subnetworks | *t* | Cohen’s *d* | *p* | adjusted *p* |
| --- | --- | --- | --- | --- |
| Visual network | 0.32 | 0.04 | 0.747 | 0.854 |
| Sensorimotor network | 3.33 | 0.45 | 0.001 | **0.004** |
| Dorsal attention network | -0.94 | -0.13 | 0.350 | 0.561 |
| Ventral attention network | 2.64 | 0.36 | 0.009 | **0.023** |
| Limbic network | 0.66 | 0.09 | 0.510 | 0.681 |
| Frontoparietal network | 0.04 | 0.005 | 0.966 | 0.966 |
| Default mode network | -3.86 | -0.53 | <0.001 | **0.001** |
| Subcortical network | 1.27 | 0.17 | 0.205 | 0.410 |

Abbreviations: adjusted *p*, FDR-adjusted *p*-values.

**Table. S3 Differences between FES and HC in the principal primary-to-transmodal gradient**

| No. | Region | x | y | z | z | Size (mm^3^) |
| --- | --- | --- | --- | --- | --- | --- |
| **FES > HC** | |  |  |  |  |  |
| 1 | Right inferior orbitofrontal gyrus, BA47 | 44 | 44 | -8 | 5.74 | 12352 |
| 2 | Left inferior orbitofrontal gyrus, BA47 | -48 | 44 | -8 | 6.37 | 4928 |
| 3 | Right putamen, BA48 | 32 | -8 | 0 | 6.58 | 10048 |
| 4 | Left putamen, BA48 | -32 | -8 | 4 | 6.08 | 8128 |
| 5 | Right cuneus, BA19 | 16 | -88 | 40 | 4.60 | 2176 |
| 6 | Left postcentral gyrus, BA4 | -44 | -16 | 48 | 4.24 | 2688 |
| **FES < HC** | |  |  |  |  |  |
| 7 | Right middle temporal gyrus, BA21 | 64 | -8 | -24 | -4.95 | 2176 |
| 8 | Left medial superior frontal gyrus, BA10 | -12 | 56 | 4 | -4.51 | 7744 |
| 9 | Left cuneus, BA23 | -16 | -60 | 24 | -6.41 | 46016 |
| 10 | Right anterior cingulate gyrus, BA32 | 12 | 44 | 4 | -3.90 | 1472 |
| 11 | Right angular gyrus, BA39 | 48 | -56 | 32 | -4.50 | 2560 |
| 12 | Left angular gyrus, BA39 | -44 | -68 | 36 | -4.69 | 2368 |
| 13 | Right middle frontal gyrus, BA9 | 24 | 24 | 44 | -4.18 | 4352 |
| 14 | Left middle frontal gyrus, BA8 | -26 | 16 | 64 | -4.21 | 6784 |

**Table. S4 Case-control differences in the global gradient metrics**

| Metric | FES, mean (SD) | HC, mean (SD) | *t* | Cohen’s *d* | *p* | adjusted *p* |
| --- | --- | --- | --- | --- | --- | --- |
| explained ratio | 0.12 (0.03) | 0.13 (0.02) | -4.20 | -0.57 | <0.001 | **<0.001** |
| range | 0.21 (0.03) | 0.23 (0.02) | -3.61 | -0.49 | <0.001 | **<0.001** |
| variance | 0.05 (0.01) | 0.06 (0.01) | -2.32 | -0.32 | 0.021 | **0.021** |
| aGamma | 0.45 (0.13) | 0.41 (0.09) | 2.66 | 0.36 | 0.008 | **0.021** |
| aLambda | 0.38 (0.05) | 0.37 (0.03) | 2.26 | 0.31 | 0.025 | **0.025** |
| aSigma | 0.35 (0.06) | 0.34 (0.05) | 2.47 | 0.34 | 0.014 | **0.021** |

Abbreviations: SD, standard deviation; aGamma, AUC of the normalized clustering coefficient; aLambda, AUC of the normalized shortest path length; aSigma. AUC of the small-world network; AUC, area under the curve; adjusted *p*, FDR-adjusted *p*-values.

**Table. S5 Spatial correlation between the meta-analytic map of cognitive terms and FES-related alterations in the primary gradient**

| FES-positive | *r* | *p* | adjusted *p* | FES-negative | *r* | *p* | adjusted *p* |
| --- | --- | --- | --- | --- | --- | --- | --- |
| Term |  |  |  | Term |  |  |  |
| language comprehension | 0.08 | 0.054 | 0.874 | memory retrieval | 0.26 | <0.001 | **0.006** |
| communication | 0.06 | 0.056 | 0.874 | autobiographical memory | 0.26 | <0.001 | **0.006** |
| meaning | 0.06 | 0.127 | 0.874 | episodic memory | 0.24 | <0.001 | **0.006** |
| language | 0.05 | 0.162 | 0.874 | retrieval | 0.23 | <0.001 | **0.006** |
| listening | 0.05 | 0.132 | 0.874 | memory | 0.18 | 0.003 | **0.031** |
| emotion | 0.04 | 0.132 | 0.874 | recall | 0.17 | <0.001 | **0.007** |
| morphology | 0.04 | 0.063 | 0.874 | semantic memory | 0.15 | 0.001 | **0.016** |
| social cognition | 0.03 | 0.182 | 0.874 | reasoning | 0.13 | <0.001 | **0.006** |
| semantic memory | 0.03 | 0.178 | 0.874 | social cognition | 0.13 | 0.004 | **0.034** |
| valence | 0.03 | 0.180 | 0.874 | intention | 0.13 | 0.001 | **0.016** |
| reading | 0.03 | 0.226 | 0.874 | psychosis | 0.12 | <0.001 | **0.006** |
| fear | 0.03 | 0.136 | 0.874 | familiarity | 0.11 | 0.005 | **0.037** |
| sentence comprehension | 0.03 | 0.189 | 0.874 | salience | 0.10 | 0.004 | **0.034** |
| context | 0.03 | 0.193 | 0.874 | belief | 0.10 | 0.015 | 0.083 |
| empathy | 0.03 | 0.229 | 0.874 | thought | 0.10 | <0.001 | **0.012** |
| integration | 0.03 | 0.233 | 0.874 | navigation | 0.10 | 0.038 | 0.179 |
| consolidation | 0.02 | 0.117 | 0.874 | knowledge | 0.10 | 0.015 | 0.083 |
| stress | 0.02 | 0.160 | 0.874 | judgment | 0.08 | 0.093 | 0.327 |
| recognition | 0.02 | 0.338 | 0.874 | inference | 0.08 | 0.016 | 0.084 |
| perception | 0.02 | 0.289 | 0.874 | goal | 0.07 | 0.042 | 0.184 |

Abbreviations: adjusted *p*, FDR-adjusted *p*-values.

**Table. S6 Differences between minor FES and adult FES in the principal primary-to-transmodal gradient**

| No. | Region | x | y | z | z | Size (mm^3^) |
| --- | --- | --- | --- | --- | --- | --- |
| **minor > adult** | |  |  |  |  |  |
| 1 | Left superior temporal gyrus, BA38 | -28 | 16 | -32 | 4.17 | 2496 |
| 2 | Right fusiform gyrus, BA37 | 32 | -44 | -20 | 5.15 | 37888 |
| 3 | Left middle temporal gyrus, BA21 | -44 | -4 | -16 | 4.40 | 6144 |
| 4 | Left pallidum, BA48 | -20 | -4 | -4 | 4.10 | 1728 |
| 5 | Right superior orbitofrontal gyrus, BA11 | 24 | 36 | -16 | 4.01 | 2844 |
| 6 | Right hippocampus, BA20 | 24 | -16 | -16 | 4.06 | 2176 |
| 7 | Right middle temporal gyrus, BA21 | 64 | 8 | -16 | 4.74 | 4800 |
| 8 | Right inferior temporal gyrus, BA44 | 48 | 12 | 32 | 4.33 | 3136 |
| 9 | Right postcentral gyrus, BA3 | 40 | -24 | 44 | 4.00 | 5248 |
| 10 | Left precentral gyrus, BA6 | -56 | 8 | 36 | 5.13 | 4160 |
| 11 | Left inferior parietal gyrus, BA3 | -52 | -20 | 40 | 4.55 | 4608 |
| 12 | Right medial cingulate gyrus, BA24 | 8 | 0 | 40 | 4.04 | 3712 |
| **minor < adult** | |  |  |  |  |  |
| 13 | Left medial superior frontal gyrus, BA10 | 0 | 64 | 12 | -5.11 | 17344 |
| 14 | Left caudate nucleus, BA48 | -20 | 16 | 12 | -4.23 | 1664 |
| 15 | Right caudate nucleus, BA48 | 16 | 24 | 12 | -4.29 | 2240 |
| 16 | Right calcarine, BA18 | 28 | -64 | 12 | -5.06 | 17344 |
| 17 | Left precuneus, BA5 | 0 | -52 | 68 | -5.06 | 11264 |
| 18 | Right medial superior frontal gyrus, BA8 | 12 | 28 | 64 | -4.95 | 11520 |
| 19 | Left middle frontal gyrus, BA8 | -36 | 16 | 60 | -4.21 | 1920 |

**Reference:**

Glasser, M. F., Coalson, T. S., Robinson, E. C., Hacker, C. D., Harwell, J., Yacoub, E., . . . Van Essen, D. C. (2016). A multi-modal parcellation of human cerebral cortex. *Nature, 536*(7615), 171-178. https://doi.org/10.1038/nature18933

Hansen, J. Y., Markello, R. D., Vogel, J. W., Seidlitz, J., Bzdok, D., & Misic, B. (2021). Mapping gene transcription and neurocognition across human neocortex. *Nature Human Behaviour, 5*(9), 1240-1250. https://doi.org/10.1038/s41562-021-01082-z

Leming, M., Su, L., Chattopadhyay, S., & Suckling, J. (2019). Normative pathways in the functional connectome. *Neuroimage, 184*, 317-334. https://doi.org/10.1016/j.neuroimage.2018.09.028

Liao, X., Vasilakos, A. V., & He, Y. (2017). Small-world human brain networks: Perspectives and challenges. *Neuroscience And Biobehavioral Reviews, 77*, 286-300. https://doi.org/10.1016/j.neubiorev.2017.03.018

Narr, K. L., & Leaver, A. M. (2015). Connectome and schizophrenia. *Current Opinion In Psychiatry, 28*(3), 229-235. https://doi.org/10.1097/yco.0000000000000157

Slinger, G., Otte, W. M., Braun, K. P. J., & van Diessen, E. (2022). An updated systematic review and meta-analysis of brain network organization in focal epilepsy: Looking back and forth. *Neuroscience And Biobehavioral Reviews, 132*, 211-223. https://doi.org/10.1016/j.neubiorev.2021.11.028
